# Deep-Learning Radiomics for Discrimination Conversion of Alzheimer's Disease in Patients With Mild Cognitive Impairment: A Study Based on ^18^F-FDG PET Imaging

**DOI:** 10.3389/fnagi.2021.764872

**Published:** 2021-10-26

**Authors:** Ping Zhou, Rong Zeng, Lun Yu, Yabo Feng, Chuxin Chen, Fang Li, Yang Liu, Yanhui Huang, Zhongxiong Huang, the Alzheimer's Disease Neuroimaging Initiative

**Affiliations:** Department of PET-CT Center, Chenzhou No.1 People's Hospital, Chenzhou, China

**Keywords:** deep learning radiomics, ^18^F-fluorodeoxyglucose positron emission tomography, mild cognitive impairment, Alzheimer's disease, classification

## Abstract

**Objectives:** Alzheimer's disease (AD) is the most prevalent neurodegenerative disorder and the most common form of dementia in the older people. Some types of mild cognitive impairment (MCI) are the clinical precursors of AD, while other MCI forms tend to remain stable over time and do not progress to AD. To discriminate MCI patients at risk of AD from stable MCI, we propose a novel deep-learning radiomics (DLR) model based on ^18^F-fluorodeoxyglucose positron emission tomography (^18^F-FDG PET) images and combine DLR features with clinical parameters (DLR+C) to improve diagnostic performance.

**Methods:** ^18^F-fluorodeoxyglucose positron emission tomography (PET) data from the Alzheimer's disease Neuroimaging Initiative database (ADNI) were collected, including 168 patients with MCI who converted to AD within 3 years and 187 patients with MCI without conversion within 3 years. These subjects were randomly partitioned into 90 % for the training/validation group and 10 % for the independent test group. The proposed DLR approach consists of three steps: base DL model pre-training, network features extraction, and integration of DLR+C, where a convolution network serves as a feature encoder, and a support vector machine (SVM) operated as the classifier. In comparative experiments, we compared our DLR+C method with four other methods: the standard uptake value ratio (SUVR) method, Radiomics-ROI method, Clinical method, and SUVR + Clinical method. To guarantee the robustness, 10-fold cross-validation was processed 100 times.

**Results:** Under the DLR model, our proposed DLR+C was advantageous and yielded the best classification performance in the diagnosis of conversion with the accuracy, sensitivity, and specificity of 90.62 ± 1.16, 87.50 ± 0.00, and 93.39 ± 2.19%, respectively. In contrast, the respective accuracy of the other four methods reached 68.38 ± 1.27, 73.31 ± 6.93, 81.09 ± 1.97, and 85.35 ± 0.72 %. These results suggested the DLR approach could be used successfully in the prediction of conversion to AD, and that our proposed DLR-combined clinical information was effective.

**Conclusions:** This study showed DLR+C could provide a novel and valuable method for the computer-assisted diagnosis of conversion to AD from MCI. This DLR+C method provided a quantitative biomarker which could predict conversion to AD in MCI patients.

## Introduction

Alzheimer's disease (AD) is the most common type of dementia. Alzheimer's disease is an irreversible, progressive neurological brain disorder expected to increase significantly in the coming years due to aging and improvement in general health care (Ferri et al., 2006; 2020 Alzheimer's disease facts figures, 2020). Because mild memory decline and cognitive deficits appear before AD clinical manifestation (Braak and Braak, 1996; Delacourte et al., 1999), increasing attention has been focused on mild cognitive impairment (MCI). As a preclinical stage of AD, MCI is a board and heterogeneous phenotypic spectrum that has no evident cognitive behavioral symptoms, but can show subtle prodromal signs of dementia (Albert et al., 2011; McKhann et al., 2011). Because of its heterogeneous presentation (Schneider et al., 2009), MCI patients may remain stable, or develop AD or other forms of dementia (Bennett et al., 2003; Sanford, 2017). Therefore, it is crucial to exploit specific risks factors and biomarkers that can predict the progression to AD from MCI.

Currently, structural and functional neuroimaging modalities, such as magnetic resonance imaging (MRI) and positron emission tomography (PET), have been used to develop biomarkers for prediction conversion to AD in patients with MCI (Brooks and Loewenstein, 2010; Vos et al., 2012; Richard et al., 2013; Lange et al., 2015; Liu et al., 2017; Zhou et al., 2019). Numerous studies using ^18^F-fluorodeoxyglucose positron emission tomography (^18^F-FDG PET) have shown that there are metabolic alterations detected in MCI patients (Caroli et al., 2012; Pagani et al., 2017). Furthermore, FDG PET was found to be the only technique that can significantly improve the predictive value of demographic covariates regarding the development of AD. It further proved to be a better predictor of conversion than MRI (Shaffer et al., 2013). Specifically, FDG PET alone has shown accuracies in predicting the progression of MCI to AD ranging between 70 and 83% (Lange et al., 2015; Liu et al., 2017; Zhou et al., 2019; Wang et al., 2020). For example, Lange et al. (2015) performed voxel-based statistical testing by the statistical parametric mapping software (SPM8) and obtained an AUC of 0.728 with default settings. Zhou et al. (2019) applied radiomics analysis methods to extract radiomic features in MCI conversion-related regions of interest (ROIs), and the accuracy of prediction reached 0.733. Liu et al. (2017) analyzed FDG PET by using independent component analysis (ICA) and Cox models to extract independent sources of information from whole-brain data, and obtained an accuracy of 0.688 in the FDG PET single modality model.

The aforementioned methods retain some limitations, however. Radiomics based on ROI depend mostly on prior knowledge. The voxel-level analysis considered information across the whole brain, but modeling based on each voxel inevitably results in heavy computing workload. Further, although ICA eliminates the need for a priori knowledge of the effects on underlying brain anatomy and uses whole-brain data, instead of a region-of-interest approach, it requires hand-coding and tedious designing processes, which is analogous to the radiomics method and voxel-level analysis.

Deep-learning radiomics (DLR), a newly developing method, can provide quantitative and high-throughput features from medical images by supervised learning (Gillies et al., 2016; Wang et al., 2019a). This algorithm implemented via deep neural networks automatically embeds computational features to yield end-to-end models that facilitate discovery of relevant highly complex feature, avoiding hand-coding, and a priori knowledge. Wang et al. (2019a) applied this DLR method to shear wave elastography images and presented excellent performances in predicting the stages of liver fibrosis. Moreover, Zheng et al. (2020) used DLR to predict axillary lymph node status in early-stage breast cancer, and clinical parameter combined DLR (DLR+C) yielded the best diagnostic performance with an AUC of 0.902. This methodology has recently extended to other medical applications, such as neurodegenerative diseases (Lu et al., 2018b; Basaia et al., 2019; Spasov et al., 2019a). However, when applied to analyze medical images, there is a scarce-sample problem with DLR. Therefore, in this study we hypothesized that the DLR method might be effective in the diagnosis of conversion to AD in patients with MCI, and DLR+C might be able to provide more valuable information and improve identification of patients likely to convert to AD. We proposed a novel computer-aided diagnosis approach for the conversion to AD from MCI, based on DLR and evaluated the diagnostic performance of DLR features combined clinical information.

## Methods and Materials

**Figure 1:**
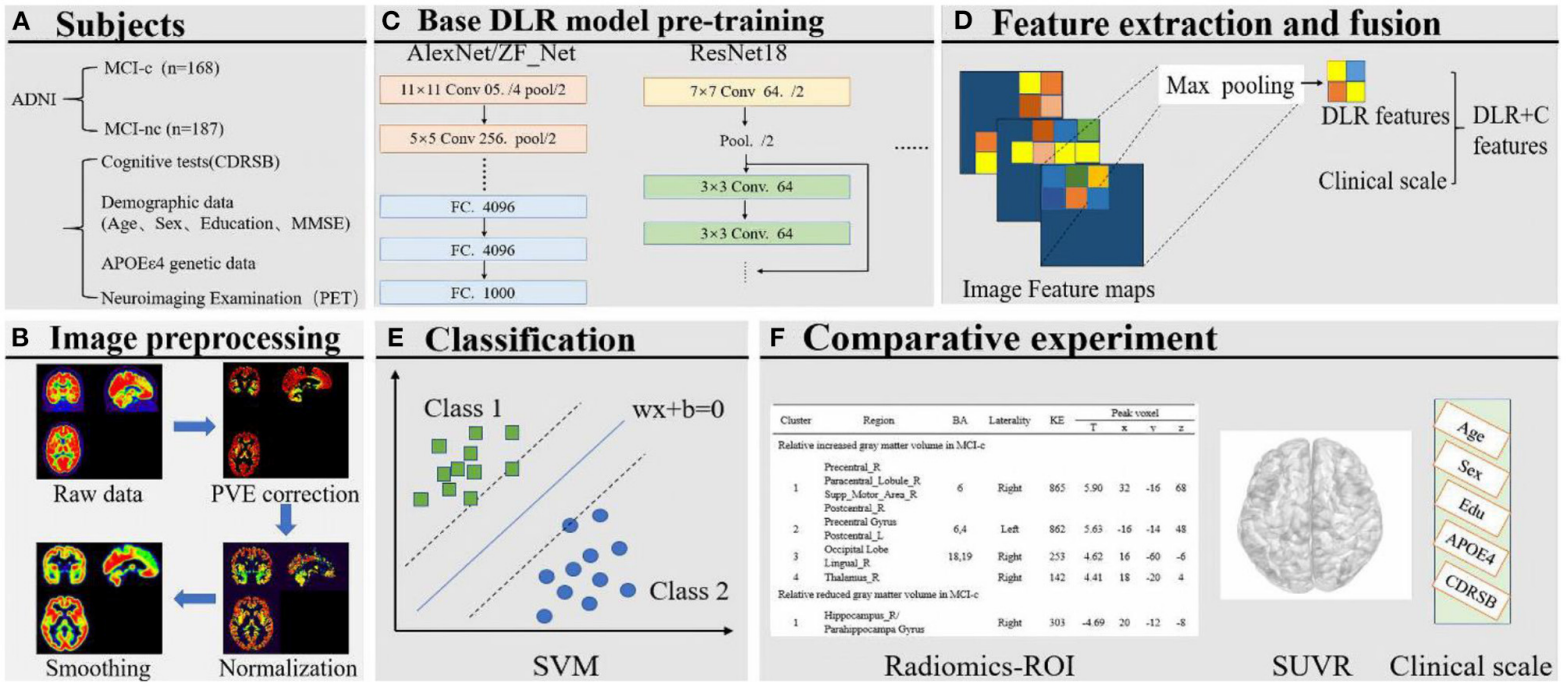
**(A)** Collection of images and clinical scales. **(B)** Image preprocessing. **(C)** Base DL model pre-training. **(D)** Feature extraction and fusion. **(E)** Classification based on SVM. **(F)** Comparative experiment.

The framework of this study, comprising six steps, is shown in Figure 1. First, we preprocessed the collected PET data, mainly including partial volume effects (PVE) correction, normalization, and smoothing. Then, several deep learning (DL) models were pre-trained to select the optimal Base DL model for DLR feature extraction. Subsequently, DLR+C were employed to classify MCI converters (MCI-c) and MCI non-converters (MCI-nc) using the Support vector machine (SVM). Simultaneously, we also designed a comparative experiment for analysis. The details are described in subsequent sections.

### Subjects

The FDG-PET image data used in the preparation of this study were obtained from the Alzheimer's Disease Neuroimaging Initiative (ADNI) database (http://adni.loni.usc.edu/). Alzheimer's Disease Neuroimaging Initiative was launched in 2003 by the National Institute on Aging, the National Institute of Biomedical Imaging and Bioengineering, the Food and Drug Administration, private pharmaceutical companies, and non-profit organizations, as a $60 million, 5-year public–private partnership. The primary goal of ADNI has devoted to test whether serial MRI, PET, other biological markers, and clinical and neuropsychological assessment can be combined to measure the progression of mild MCI and early AD. Up-to-date information is provided on http://www.adni-info.org.

In this study, we collected 168 MCI-c and 187 MCI-nc PET Scan data from ADNI 1, ADNI 2, and ADNI GO cohorts in the ADNI database. Eligible participants with MCI underwent FDG-PET scanning and clinical cognitive evaluations at the baseline and were clinically followed-up during at least 36 months. Detailed eligibility criteria for these participants are as follows: (1) For MCI-nc, participants were evaluated for at least 3 years (including a 3 year time point) from the time of initial data collection. Scan data for MCI-nc were collected at baseline 3 and these participants did not convert to AD during the 3 years follow-up period. (2) For MCI-c, the evaluation time may be less than 3 years. Scan data for MCI-c were not all collected at the baseline. Participants with a bidirectional change of diagnosis (MCI to AD, and back to MCI) within the follow-up period were excluded.

All subjects were divided into two groups, a Training & Validation Group and an independent test group. Our Training & Validation Group contained 152 subjects with MCI-c, and 169 MCI-nc subjects. We used the FDG-PET scan data from this group to establish and test the validity of our predictive models. Our test group consisted of 16 MCI-c subjects and 18 MCI-nc subjects, and it was used to evaluate the diagnostic value of the predictive models. Demographic data including age, gender, sex, education, and neuropsychological cognitive assessment tests including the dementia rating scale (CDRSB), as well as the apolipoprotein E (APOE) ε4 genotyping characteristics of the dataset, are shown in Table 1.

**Table 1:** Demographic and statistics of clinical assessments at time of data collection.

**Groups**	**Gender (M/F)**	**Age (years)**	**EDU**	**MMSE**	**MoCA**	**APOEε4 positive rate**	**CDRSB**
**Training/Validation Groups**							
MCI_c (*n* = 152)	86/66	74.2 ± 7.0	16.0 ± 2.7	26.5 ± 2.2	21.0 ± 2.9	65.1%	2.4 ± 1.0
MCI_nc (*n* = 169)	96/73	72.2 ± 7.4^b^	16.1 ± 2.6	28.1 ± 1.6^b^	23.9 ± 2.5^b^	34.9%	1.2 ± 0.7^b^
**Test Groups (*****n*** **=** **48)**							
MCI_c (*n* = 16)	9/7	71.4 ± 7.8	16.3 ± 2.5	26.3 ± 2.0	21.5 ± 2.1	75.0%	2.5 ± 1.1
MCI_nc (*n* = 18)	13/5^a^	71.3 ± 8.7	15.8 ± 2.8	27.7 ± 1.8^b^	23.2 ± 3.8	44.4%	0.9 ± 0.6^b^

*All data except APOEε4 positive rate were presented as mean ± standard deviation. EDU, education; MMSE, Mini-mental State Examination; MoCA, Montreal Cognitive Assessment; CDRSB: clinical dementia rating sum of boxes*.

a,b *Group-level two-sample t test are conducted for Age, Education, MMSE, MoCA, and CDRSB; Group-level chi-square test are conducted for Gender*.

### FDG-PET Images Acquisition and Preprocessing

The PET acquisition process is detailed in the online information of the ADNI project. In 290 cases, dynamic 3D scans with six 5-min frames were acquired 30 min after injection of 185 ± 18.5 MBq FDG, and all frames were motion-corrected to the first frame and then summed to create a single image file. In the remaining cases (*n* = 65), patients were scanned for a static 30-min acquisition period.

Individual PET scan preprocessing (Ding et al., 2021; Dong et al., 2021) was performed by statistical parametric mapping (SPM12) software (Wellcome Department of Imaging Neuroscience, Institute of Neurology, London, United Kingdom) using Matlab2016b (Mathworks Inc., Sherborn, MA, USA). First, PET images were co-registered with their corresponding T1-weighted images and then corrected for PVE based on the Muller–Gartner algorithm, where PVE correction was applied to the images to minimize the PVE on PET measurements (Gonzalez-Escamilla et al., 2017). Thereafter, through linear and non-linear 3D transformations, the images were spatially normalized to a PET template in the Montreal Neurological Institute (MNI) brain space. The normalized PET images were then smoothed by a Gaussian filter of 8 mm full-width at half-maximum (FWHM) over a 3D space to blur the individual anatomical variations and to increase the signal-to-noise ratio for subsequent analysis. Finally, individual PET images were intensity normalized to the global mean brain uptake and automatically parcellated into 90 ROIs defined by the automated anatomical labeling (AAL) atlas. The processed images had a spatial resolution of 91 × 109 × 91 with a voxel size of 2 × 2 × 2 mm^3^. Lastly, each three-dimensional PET image was sliced and tiled into two-dimensional images, then being resized to 224 ^*^ 224 pixels for subsequent DL model pre-training.

### Deep-Learning Radiomics Model

**Figure 2:**
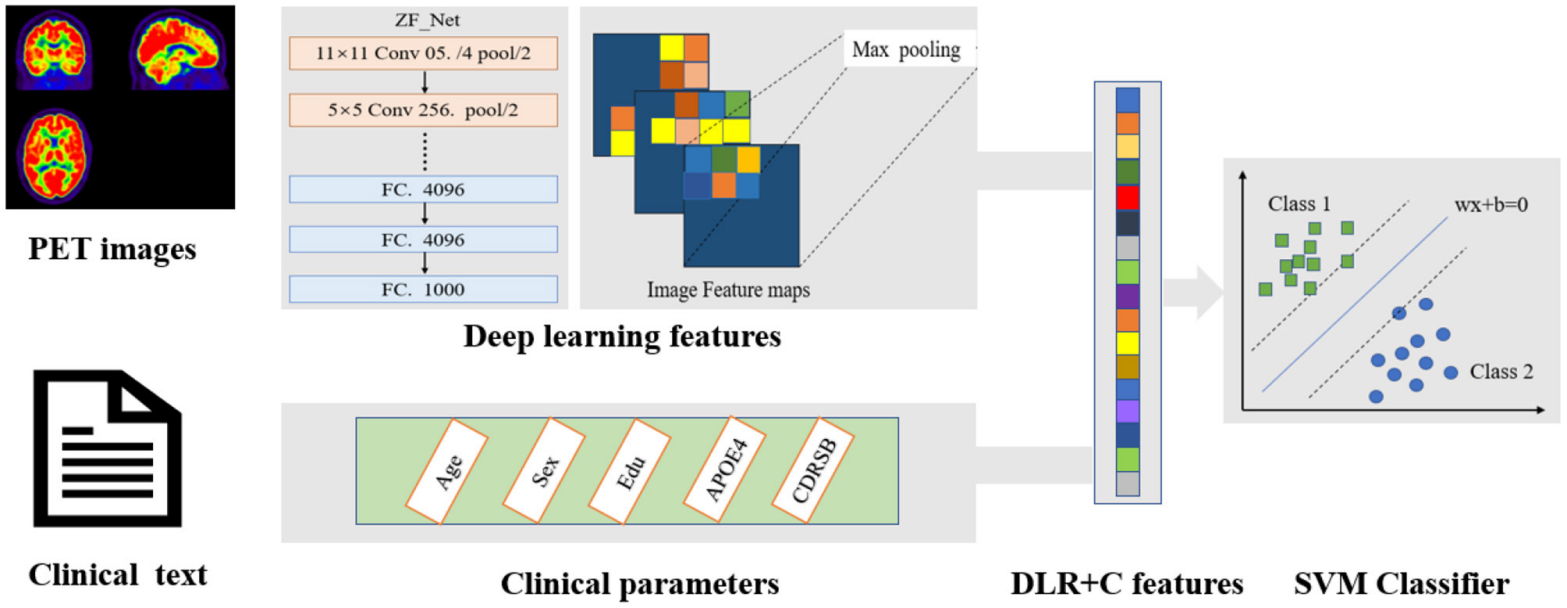
The overall pipeline of DLR model. The pre-trained ZF-Net model acted as an feature encoder of the input images. Then the DLR features combined clinical parameters were classified by a SVM classifier.

Figure 2 shows the pipeline of our proposed DLR method. The method is composed of three steps: (1) Base DL model pre-training, where we pre-trained several DL models and chose the optimal as the final DL model, to extract high-throughput DLR features of PET images; (2) Feature Fusion; and (3) Classification. Based on aforementioned DLR fusion features, SVM was used as the classifier to discriminate conversion to Alzheimer's disease in patients with MCI. Detailed technical demonstrations are described in the following sections.

### Base DLR Model Pre-training

The Base DLR model acts as a feature encoder, which has a significant impact on classification. In this study, five convolutional neural networks (CNNs) namely AlexNet, ZF-Net, ResNet18, InceptionV3, and Xception, were introduced for pre-training to find the most suitable model for identifying conversion to AD from stable MCI patients.

In general, the complexity of the CNNs depends on two factors, namely “depth” and “width.” The advantage of DL is that it can learn more representative features with the help of its neural network with numerous layers and broad width. But DL is flawed with highly dependence on data. Consequently, deeper networks do not necessarily reach better performance. This is mainly because the multi-layer back propagation of the error signal can easily lead to the gradient “dispersion” or the gradient “disappears” (He et al., 2016), based on the stochastic gradient descent when training. Especially for the sparse sample characteristics of medical images, the deeper network performs poorly, leading to overfitting. Considering above factors, to compare model performance, we introduced five CNNs, specifically AlexNet and ZF-Net with simple network structures, ResNet18, InceptionV3, and Xception with more network layers.

AlexNet, containing five convolutional layers and three fully-connected layers with learnable weights, competed in the ImageNet challenge in 2012 and achieved a top-five error of only 15.3% (Wang et al., 2019b; Rehman et al., 2020). There are several advanced techniques in AlexNet compared with traditional neural networks, including employing the rectified linear unit (ReLU) function and a pool operation. ZF-Net is based on AlexNet with only some changes in the convolutional kernel and step size, with no significant breakthrough in the network structure. Instead, based on the traditional CNN framework, the network structure of InceptionV3, Xception, and ResNet18 are more complex and deeper, and have their own unique network characteristics. The greatest advantage of the ResNet framework lies in adding identity mapping that is performed by the shortcut connections, the outputs of which are added to the outputs of the stacked layers (Chen et al., 2019). Therefore, the ResNet addressed the degradation problem and added neither extra parameters nor computational complexity. The advantage of Google's Inception structures is that there are good performance especially under strict constraints on memory and complexity of computational problems (Khosravi et al., 2018). For example, GoogLeNet (Szegedy et al., 2015) used five million parameters and the amount of parameters has significant reduction when compared with AlexNet (Krizhevsky et al., 2017). For this, Inception networks are always chose when a huge of data need to be processed at reasonable time and computational cost. And Inception V3 is one version of attempts to scale up deep networks, in which the fully connected layer of the auxiliary classifier is also-normalized based on Inception V2. In addition, Xception is an improved model based on Inception V3, whose main improvement is to use depth wise separable convolution to replace the Inception module.

There were two steps included in the entire training process, the forward computation and the backward propagation. Before modeling, the three-dimensional PET image of each subject was sliced and tiled into two-dimensional images, then being resized to 224 × 224 pixels and normalized. The pathology type was encoded to one hot, which was the label. Thereafter, in the training stage, data was fed into the network to update model parameters via backward propagation with the SGD algorithm, a first-order gradient-based optimization algorithm that has been proven to be computationally efficient and appropriate for training deep neural networks. The outputs of the network were used as classification results, and the cross-entropy of the outputs was calculated as the loss function. More specifically, the output of the network for each individual PET image could be a binary value, in which one represented the highest probability of being MCI-c subjects, while zero represented highest probability of being MCI-nc subjects.

We employed several DL frameworks in this study. In the pre-training, we set the learning rate into 1e−2 and applied the SGD optimizer to update model parameters with a batch size of 8. The maximum number of iterations was set into 100. Note that we used Dropout and Early Stopping in this step to alleviate overfitting of our models, and we also adopted a learning rate decay strategy, setting the learning rate decay step to 10. Furthermore, a strategy called online data augmentation was used to prevent overfitting of small datasets, which meant horizontal flipping and Gaussian noise addition for input images in the training/validation group. Above all, pre-training of deep-learning models was processed on a GPU (graphics processing unit, GTX 1080 Ti acceleration of PyCharm 3.5).

### DLR Features

Contrasting with hand-crafted and engineered features designed in previous medical experiences, DLR learned the high-throughput image features in a supervised manner, which could make full use of embedded information in PET images. After screening the optimal Base DL model, we replaced the FC layer with an SVM as classifier and fused the clinical information and network features to collaboratively make decisions.

Specifically, to obtain DLR features, the feature maps were first extracted from the last convolution layer of the convolution network, and they were transformed to raw values by taking the maximum values of each feature map with global max pooling. Afterwards, these extracted features, defined as DLR features, were combined with clinical parameters (CDRSD, Age, MMSE, etc.) as input data for future classification.

### Classification

In this study, the enrolled subjects were randomly divided into one training/validation group and one independent test group at a ratio of 9:1, as shown in Table 1. The training group was then used to optimize the model parameters. We also randomly chose 10% of the training group to form a validation group to guide the choice of hyper parameters. We conducted training of several deep-learning models, including AlexNet, ZF-Net, ResNet18, InceptionV3, and Xception, and compared the classification performance for screening the optimum DLR. To evaluate classification performance, we repeatedly conducted 10-fold cross-validation in the training group. Subsequently, the extracted DLR features were combined with clinical scales, which were together named as DLR+C features serving as input. SVM served as a classifier to perform the classification. The training/validation group was used to train and validate the model, while the test group was used as an independent test dataset to verify the predictive performance of our proposed DLR+C approach. The model was trained and validated with 10-fold cross-validation 100 times. The linear kernel function was used to detect feature generalization ability and classification reliability.

The mean [± standard deviation (SD)] accuracy, sensitivity, and specificity were used to evaluate the results. The mathematical expression of the three parameters was as follows:


(1) $$\begin{array}{r} \begin{array}{c} Accuracy=\;\frac{Tn+Tp}{Tn+Tp+Fn+Fp}\\ Sensitivity=\;\frac{Tp}{Tp+Fn}\\ Specificity=\;\frac{Tn}{Tn+Fp} \end{array} \end{array}$$

where *Tn, Tp, Fn*, and *Fp* denote true negatives, true positives, false negatives, and false positives, respectively.

Simultaneously, a receiver operating characteristic (ROC) curve was produced to intuitively compare the results of the different approaches, and the area under the curve (AUC) of the ROC was computed to quantitatively evaluate classification performance.

### Comparative Experiment

To verify the superiority of the proposed DLR+C method in this research, we deployed the following four comparative experiments. They were all built with SVM classifiers, but with different input data. (1) Radiomics method: radiomic features of ROI in the brain (Supplementary Material 1, Zhou et al., 2019); (2) Standard uptake value ratio (SUVR) method: mean voxel uptake ratio of the whole brain according to AAL template; (3) Clinical method: Demographic data, neuropsychological cognitive assessment tests, as well as the APOE ε4 genotyping characteristics of all subjects. (4) SUVR + Clinical method.

Likewise, during the comparative experiments, the 10-fold cross-validation was performed in the training/validation group with 100 repetitions with the linear kernel. The test group was used to independently verify the generalization ability of the above model.

### Decision Score

To more efficiently describe the discrimination ability of our proposed DLR+C method, we conducted a statistical analysis of the decision scores. A decision score could be output after the SVM model decision analysis to represent the class scores of MCI-nc or MCI-c. In the experiment, we calculated separately the decision scores of MCI-nc and MCI-c subjects of the test group. We used the scores to perform the *t*-test between MCI-nc and MCI-c to observe intergroup differences.

### Statistical Analysis

Demographic and clinical characteristics were compared between groups using a two-sample *t*-test or the chi-square test. All statistical analyses were performed using SPSS Version 22.0 software (SPSS Inc., Chicago, IL, USA) and Matlab2016b (Mathworks Inc., Sherborn, MA, USA). All *p*-values < 0.05 were considered significant.

## Results

### Base DLR Model Selection

**Figure 3:**
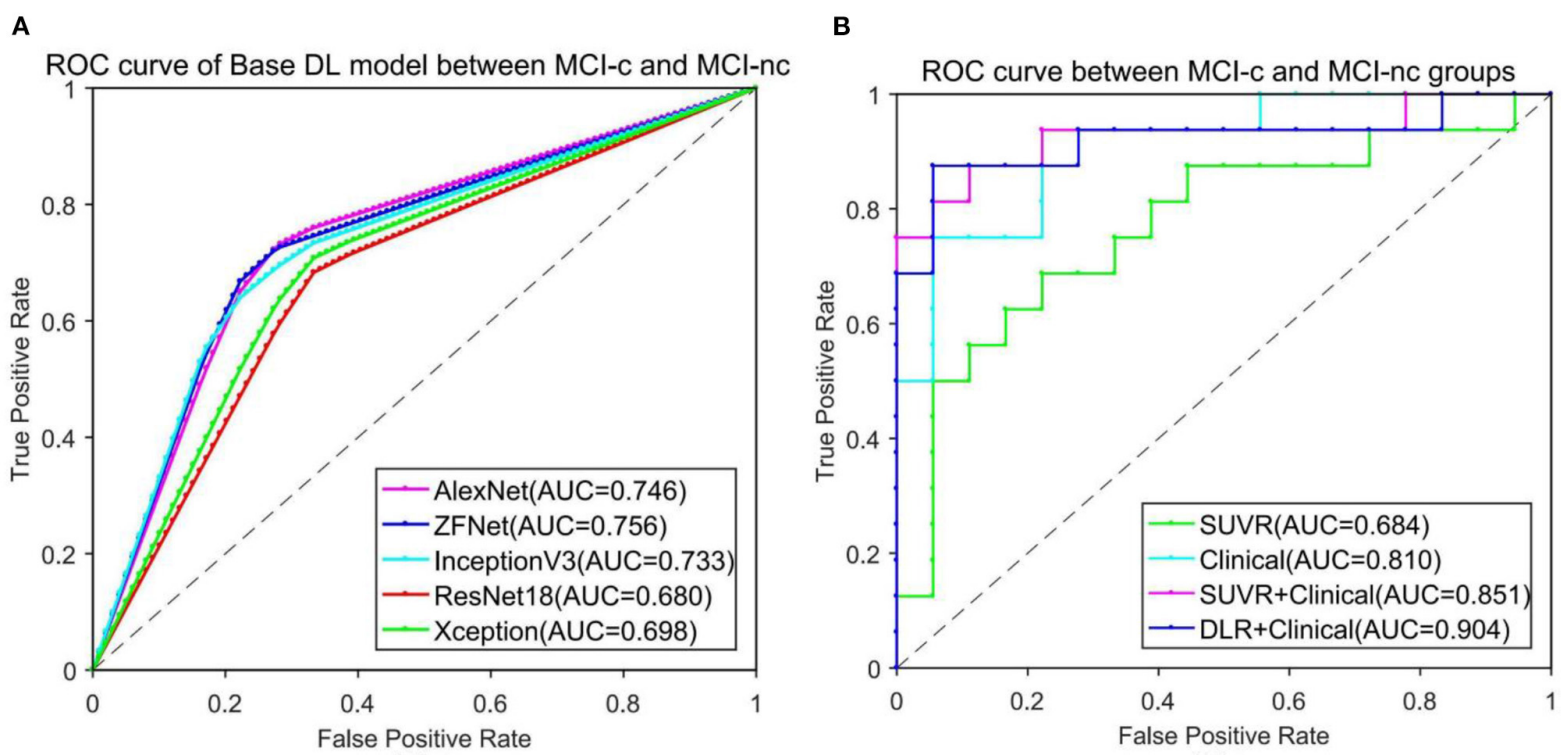
ROC curve comparison in classification of MCI-c and MCI-nc. **(A)** ROC curve of five different Base DL pre-training models. **(B)** ROC curve of four different comparative experiments.

To find the suitable Base DLR model for MCI-c vs. MCI-nc classification, the performances of AlexNet, ZF-Net, ResNet18, InceptionV3, and Xception in classifying MCI categories were compared. The classification performances on AlexNet, ZF-Net, ResNet18, InceptionV3, and Xception models are summarized in Table 2, including the classification accuracy, sensitivity, specificity, AUC, and execution time. Specially, the accuracy, sensitivity, specificity, AUC, and execution time of the ZF-Net were 74.12 ± 2.32, 70.63 ± 3.02, 77.22 ± 4.10%, 0.756, and 231.20 s, respectively. Finally, among these five models, the ZF-Net model proved to be the suitable model which not only had the best classification performance in the independent test group, but also had a shorter model training time. Therefore, ZF-Net was selected as the basic model to extract DLR features for further study. The ROC curves of the DLR pre-training models in the classification of MCI-c and MCI-nc were presented in Figure 3A.

**Table 2:** Performance of different classification approaches in mutiltasking classification.

**Model**	**Accuracy (%)**	**Sensibility (%)**	**Specificity (%)**	**AUC**	**Execution time (s)**
AlexNet	74.11 ± 2.88	73.12 ± 2.86	75.00 ± 4.48	0.746 ± 0.03	225.20 ± 72.59
**ZF-Net**	**74.12** **±** **2.32**	**70.63** **±** **3.02**	**77.22** **±** **4.10**	**0.756** **±** **0.04**	**231.20** **±** **69.56**
InceptionV3	73.53 ± 4.60	69.37 ± 6.22	77.22 ± 6.65	0.733 ± 0.05	1090.00 ± 278.2
ResNet18	67.94 ± 2.92	68.75 ± 4.17	67.22 ± 3.15	0.680 ± 0.03	330.40 ± 55.71
Xception	69.71 ± 3.68	70.63 ± 4.22	68.89 ± 4.68	0.698 ± 0.04	665.50 ± 174.70

*The methods are conducted with 10-fold cross-validation and their results are given as mean ± standard deviation*.

*Bold values indicate classification results of the optimal model ZFNet for Base DLR Model Selection*.

### Clinical Information Integration

When ZF-Net was selected as the Base DLR model with the best performance, clinical information was added to the diagnostic model. Consistently with the method described above, the model incorporating clinical information was called ZF-Net + C, where C represents clinical information. The method of adding clinical information was to directly fuse clinical information with the extracted DLR features from the last convolution layer of the convolution network. Thereafter, the fused features were fed into the SVM classifier. The ZF-Net + C, which integrated the deep features and clinical information offline, proved to be the best in terms of classification performance. Detailed results are summarized in Table 3.

**Table 3:** Performance of different classification approaches in mutiltasking classification.

**Method**	**Accuracy (%)**	**Sensibility (%)**	**Specificity (%)**	**AUC**
SUVR method	68.38 ± 1.27	68.06 ± 0.00	68.40 ± 2.41	0.68 ± 0.01
Radiomics-ROI	73.31 ± 6.93	–	–	–
Clinical method	81.09 ± 1.97	75.69 ± 3.19	85.89 ± 4.63	0.81 ± 0.02
SUVR+Clinical	85.35 ± 0.72	81.13 ± 0.88	89.11 ± 1.09	0.85 ± 0.01
**Our proposed DLR+C**	**90.62** **±** **1.16**	**87.50** **±** **0.00**	**93.39 ± 2.19**	**0.90** ± **0.01**

*The methods are conducted with cross-validation and their results are given as mean ± standard deviation. DLR+C, Deep learning radiomics combined Clinical parameters; ROI, regions of interest*.

*Bold values represent the classification performance of the proposed DLR+C method*.

### Classification Performance

Table 3 lists the detailed results of five different methods including the SUVR method, Radiomics-ROI method, Clinical method, and DLR+C method in classification of MCI-c and MCI-nc subjects. Among five methods, the DLR+C method showed the best performance with accuracy of 90.62 ± 1.16%, sensibility of 87.50 ± 0.00%, and specificity of 93.39 ± 2.19% in the independent test group. The performance of the SUVR method, radiomics method, clinical method, and SUVR+Clinical method were all poorer than our proposed method, with accuracies of 68.38 ± 1.27, 73.31 ± 6.93, 81.09 ± 1.97, and 85.35 ± 0.72% in the independent test group, respectively.

Figure 3B presents the ROC curves of the five models in classification of MCI-c and MCI-nc. The average AUCs (±SD) of SUVR method, Clinical method, SUVR+Clinical method, and DLR+C method were 0.68 ± 0.01, 0.81 ± 0.02, 0.85 ± 0.01, and 0.90 ± 0.01, respectively, in the independent test group.

### Decision Score

**Figure 4:**
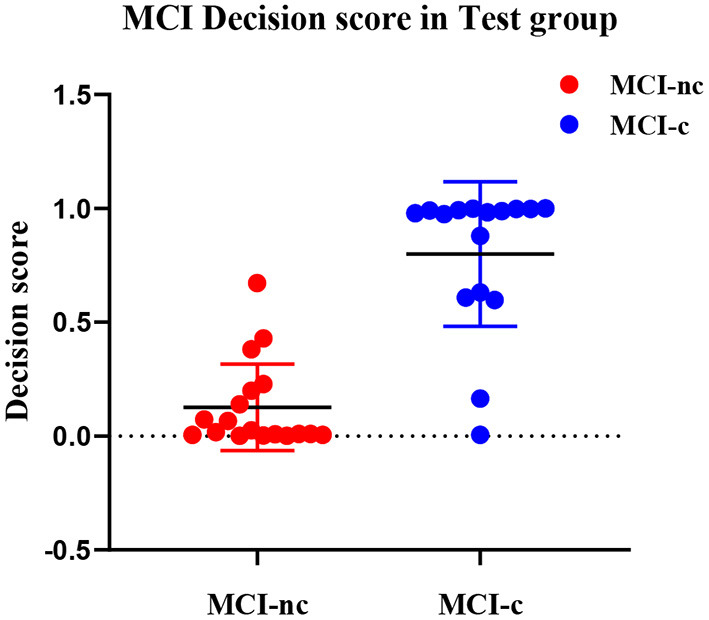
The distribution of decision score of MCI-c and MCI-nc subjects.

In our proposed DLR+C method, the performance of the output decision scores with the SVM linear kernel classification in the test group is shown in Figure 4. Decision scores of MCI-c were significantly higher than those of MCI-nc (linear: 0.82 ± 0.32 vs. 0.11 ± 0.19, *P* < 0.001). The results indicate that decision scores from the SVM output could effectively classify MCI-c and MCI-nc with significant differences, and could be used as a quantitative biomarker for classification between MCI-nc and MCI-c groups.

## Discussion

In this study, we proposed and applied a DLR+C method based on ^18^F-FDG PET images to predict conversion to AD from stable MCI. Compared with other four traditional methods including Radiomics-ROI method, Clinical model or the voxel-level analysis, our proposed DLR+C model showed significant superiority in classification of MCI-nc and MCI-c subjects, demonstrating that the DLR+C model can be used for effectively learning superior feature representation from small neuroimging data and avoid hand-coding and ROI segmentation based on a prior knowledge. Further, we validated that DLR+C had the potential to serve as a quantitative biomarker through the statistical analysis of decision scores. Overall, DLR+C might have possibility to provide clinicians with directions for the diagnosis of conversion to AD from stable MCI.

### Base DLR Model Selection

As an emerging technique for image quantitative analysis, the DLR method represents a combination and development of DL and radiomics. The DLR method can automatically learn a large number of features including a neural network's hidden layers according to input images, and this process do not require object segmentation and hard-coded feature extraction (Lu et al., 2018b; Basaia et al., 2019; Spasov et al., 2019a; Roy et al., 2020; Yee et al., 2020; Pan et al., 2021). This has been successfully applied to oncology and cancer diagnosis at the present (Han et al., 2017; Deepak and Ameer, 2019; Jeyaraj and Samuel Nadar, 2019). In this study, DLR adopted CNN frameworks and was completely established on the analysis of 2D-slice FDG PET images.

To construct a DLR feature encoder, we compared the performance of several CNN models, including AlexNet, ZF-Net, ResNet18, InceptionV3, and Xception. As shown in Table 2, we observed that the results of ZF-Net were superior to those of other CNNs, showing the mean ± SD accuracy of 74.12 ± 2.32% in the independent test group. Further, in the process of training the model, with its simple network structure and fewer model parameters, the ZF-Net model exhibited a significantly shorter training time than other models, which was what we expected. Therefore, we chose ZF-Net as the final DLR model and feature encoder. The classification result was consistent with that of Yee et al. (2020) which used a 3D CNN with residual connections that took a 3D FDG-PET image as input and obtained an accuracy rate of 0.747. It was worth nothing that Yee et al. enrolled 871 MCI-nc and 362 MCI-c participants, but participants in our study were much fewer and also achieved the same performance. Besides, there are indeed advantages about the ResNet18, InceptionV3, and Xception networks. But their classification results were still poor when the execution time became longer, which was not what we expected. We speculated it might be due to too few subjects in our study which did not matched with deeper network structures and led to overfitting.

In addition, the above process about Base DLR model selection was also repeated when not resizing images after standard preprocessing. We found similar classification performance, but the later has heavier GPU load. Therefore, the results based on sliced and resized 224^*^224 images were taken as final.

### Clinical Integration and Classification Performance

One issue is that a data scarcity problem remains when DLR is applied in medical databases (Dluhoš et al., 2017). Insufficient inputs proved incapable of training effective network parameters, and thus the optimal model becomes elusive. Considering this, we proposed the DLR+C method, providing complementary information to improve the diagnosis of conversion to AD.

According to the classification results of Table 3, our proposed DLR+C method obtained the mean accuracy of 90.62% and outperformed the result of the Base DL model. Hence, the ^18^F-FDG PET images after integrating with standard cognitive tests (CDRSB), demographic information (age, gender, education, and MMSE), and APOEε4 genetic status indeed represented more valuable information and thus improved the diagnostic performance. Further, as discussed in the study of Moradi et al. (2015), the diagnostic labeling and number of ADNI subjects vary across studies, thus impeding direct comparison. Hence, to validate the superiority of our DLR+C method, we designed comparative experiments at three levels in turn: the voxel-based, radiomics, and the clinical. As shown in Table 3, the voxel-level analysis, SUVR method, performed the poorest with a mean accuracy of 68.38%. The Clinical method obtained a mean accuracy of 81.09%, and the SUVR+Clinical method had an accuracy of 85.35%. These results were consistent with previously relevant publications, where data were collected from the ADNI database (Young et al., 2013; Liu et al., 2017; Spasov et al., 2019b), and thus verified the validity and reliability of our experiments. Young et al. (2013) used the voxel-based method and obtained 69.9% accuracy, 55.3% sensitivity, 77.1% specificity by SVM classifiers. Moreover, the results of our clinical method were coherent with those in Spasov et al. (2019b) and Liu et al. (2017), where clinical data were provided with demographic information, cognitive tests, and APOEε4 status. Spasov et al. (2019b) achieved 81% accuracy, 83% sensitivity, 81% specificity; Liu et al. (2017) achieved 81.62% accuracy, 77.78% sensitivity, and 86.11% specificity. Nonetheless, it is deserving to clarify that the outcome of our proposed DLR+C method is optimal. In summary, the above results sufficiently illustrated the superiority of our DLR+C method. DLR avoided the need for prior knowledge and hard-coded feature extraction, while clinical parameters provided more complementary and valuable information.

### Decision Score

To better demonstrate the discriminability of the proposed DLR+C method, we conducted a statistical analysis of decision values. As the distribution of decision scores in Figure 4, there were significant differences of decision scores between MCI-c and MCI-nc groups. Thus, it could be used as a quantitative biomarker for classification between the MCI-nc and MCI-c groups.

## Limitations

Although the DLR+C method enhanced the performance of discrimination of conversion to AD in patients with MCI, some limitations must be addressed. First, we need more available data to verify the generalizability and robustness of the proposed method. In this study, a small number of subjects were collected only from the ADNI database. Although the final DLR+C model performed excellent diagnostic performance, there is still potential to improve the representation of our Base DL model, where the accuracy only reached 74.12% in the independent test group and did not exceed those of Pan et al. (2021) and Lu et al. (2018a). Therefore, it is possible to improve the performance of our DLR+C method when comprehensive and homogeneous databases are developed and become available. Secondly, in this study, the DLR+C method was focused on the single image modality of ^18^F-FDG PET. Whether multi-modalities of ^18^F-FDG PET combined MRI can improve the classification performance of DLR+C method is to be explored in a further study. Third, the proposed method can provide a prediction whether MCI subjects would convert to AD, but it cannot decide when the conversion occurs in the future. To enroll longitudinal data to determine the severity of MCI-c subjects may well be of interest in our following studies.

## Conclusion

We developed a DLR+C method for the ^18^F-FDG PET modality in an effort to perform the diagnosis of MCI-c and MCI-nc subjects. This study demonstrates that the proposed DLR+C method can improve the diagnostic performance and provide a quantitative biomarker for predicting conversion to AD in MCI patients. Future, the DLR+C model holds potential to become a practical method for the computer-assisted diagnosis of conversion to AD. Prospective multi-modalities research is expected to apply our proposed DLR+C method and acquire more reliable evidence in predicting the conversion of MCI to AD.

## Data Availability Statement

The original contributions presented in the study are included in the article/Supplementary Material, further inquiries can be directed to the corresponding author/s.

## Ethics Statement

Ethical review and approval was not required for the study on human participants in accordance with the local legislation and institutional requirements. The patients/participants provided their written informed consent to participate in this study. Written informed consent was obtained from the individual(s) for the publication of any potentially identifiable images or data included in this article.

## Author Contributions

PZ, RZ, and ZH conceived and designed the experiments, analyzed and interpreted the data, and wrote the manuscript. LY, FL, and CC performed the experiments and wrote the manuscript. CC, YF, YL, and YH analyzed and interpreted the data and wrote the manuscript. The Alzheimer's Disease Neuroimaging Initiative contributed reagents, materials, and data. All authors contributed to the article and approved the submitted version.

## Funding

This program is sponsored by scientific development projects from ChenZhou Municipal Science and Technology Bureau (No.yfzx201906). Data collection and dissemination for this project were funded by the Alzheimer's Disease Neuroimaging Initiative (ADNI): the National Institutes of Health (grant number U01 Hindawi Template version: Apr19 14 AG024904), and the Department of Defense (award numberW81XWH-12-2-0012). ADNI is funded by the National Institute of Aging and the National Institute of Biomedical Imaging and Bioengineering as well as through generous contributions from the following organizations: AbbVie, Alzheimer's Association, Alzheimer's Drug Discovery Foundation, Araclon Biotech, BioClinica Inc., Biogen, Bristol-Myers Squibb Company, CereSpir Inc., Eisai Inc., Elan Pharmaceuticals Inc., Eli Lilly and Company, EuroImmun, F. Hoffmann-La Roche Ltd. and its affiliated company Genentech Inc., Fujirebio, GE Healthcare, IXICO Ltd., Janssen Alzheimer Immunotherapy Research & Development LLC., Johnson & Johnson Pharmaceutical Research &Development LLC., Lumosity, Lundbeck, Merck & Co. Inc., Meso Scale Diagnostics LLC., NeuroRx Research, Neurotrack Technologies, Novartis Pharmaceuticals Corporation, Pfizer Inc., Piramal Imaging, Servier, Takeda Pharmaceutical Company, and Transition Therapeutics. The Canadian Institutes of Health Research are providing funds to support ADNI clinical sites in Canada. Private sector contributions are facilitated by the Foundation for the National Institutes of Health (www.fnih.org). The grantee organization is the Northern California Institute for Research and Education, and the study is coordinated by the Alzheimer's Disease Cooperative Study at the University of California, San Diego, CA, USA. ADNI data are disseminated by the Laboratory for Neuro Imaging at the University of Southern California, CA, USA.

## Conflict of Interest

The authors declare that the research was conducted in the absence of any commercial or financial relationships that could be construed as a potential conflict of interest.

## Publisher's Note

All claims expressed in this article are solely those of the authors and do not necessarily represent those of their affiliated organizations, or those of the publisher, the editors and the reviewers. Any product that may be evaluated in this article, or claim that may be made by its manufacturer, is not guaranteed or endorsed by the publisher.

## References

[B1] 2020 Alzheimer's disease facts and figures (2020). Alzheimers Dement. 16, 391–460. 10.1002/alz.12068 32157811

[B2] Albert M. S. DeKosky S. T. Dickson D. Dubois B. Feldman H. H. Fox N. C.. (2011). The diagnosis of mild cognitive impairment due to Alzheimer's disease: recommendations from the National Institute on Aging-Alzheimer's Association workgroups on diagnostic guidelines for Alzheimer's disease. Alzheimers Dement. 7, 270–279. 10.1016/j.jalz.2011.03.008 21514249 PMC3312027

[B3] Basaia S. Agosta F. Wagner L. Canu E. Magnani G. Santangelo R.. (2019). Automated classification of Alzheimer's disease and mild cognitive impairment using a single MRI and deep neural networks. NeuroImage Clin. 21, 101645. 10.1016/j.nicl.2018.101645 30584016 PMC6413333

[B4] Bennett D. A. Wilson R. S. Schneider J. A. Evans D. A. Mendes de Leon C. F. Arnold S. E.. (2003). Education modifies the relation of AD pathology to level of cognitive function in older persons. Neurology 60, 1909–1915. 10.1212/01.WNL.0000069923.64550.9F 12821732

[B5] Braak H. Braak E. (1996). Development of Alzheimer-related neurofibrillary changes in the neocortex inversely recapitulates cortical myelogenesis. Acta Neuropathol. 92, 197–201. 10.1007/s004010050508 8841666

[B6] Brooks L. G. Loewenstein D. A. (2010). Assessing the progression of mild cognitive impairment to Alzheimer's disease: current trends and future directions. Alzheimer's Res. Ther. 2, 28. 10.1186/alzrt52 20920147 PMC2983437

[B7] Caroli A. Prestia A. Chen K. Ayutyanont N. Landau S. M. Madison C. M.. (2012). Summary metrics to assess Alzheimer disease-related hypometabolic pattern with 18F-FDG PET: head-to-head comparison. J. Nucl. Med. 53, 592–600. 10.2967/jnumed.111.094946 22343502 PMC3640308

[B8] Chen K. J. Chen K. L. Wang Q. He Z. Y. Hu J. He J. L. (2019). Short-term load forecasting with deep residual networks. IEEE Trans. Smart Grid 10, 3943–3952. 10.1109/TSG.2018.2844307

[B9] Deepak S. Ameer P. M. (2019). Brain tumor classification using deep CNN features via transfer learning. Comput. Biol. Med. 111, 103345. 10.1016/j.compbiomed.2019.103345 31279167

[B10] Delacourte A. David J. P. Sergeant N. Buée L. Wattez A. Vermersch P.. (1999). The biochemical pathway of neurofibrillary degeneration in aging and Alzheimer's disease. Neurology 52, 1158–1165. 10.1212/WNL.52.6.1158 10214737

[B11] Ding C. Zhang Q. Wang L. (2021). Coupling relationship between glucose and oxygen metabolisms to differentiate preclinical Alzheimer' s disease and normal individuals. Hum. Brain Mapp. 2021, 1–12. 10.1002/hbm.25599 34291850 PMC8449101

[B12] Dluhoš P. Schwarz D. Cahn W. van Haren N. Kahn R. Španiel F.. (2017). Multi-center machine learning in imaging psychiatry: a meta-model approach. Neuroimage 155, 10–24. 10.1016/j.neuroimage.2017.03.027 28428048

[B13] Dong Q. Li T. Jiang X. Wang X. Han Y. Jiang J. (2021). Glucose metabolism in the right middle temporal gyrus could be a potential biomarker for subjective cognitive decline : a study of a Han population. Alz. Res. Therapy 13, 74. 10.1186/s13195-021-00811-w 33827675 PMC8028241

[B14] Ferri C. P. Prince M. Brayne C. Brodaty H. Fratiglioni L. Ganguli M.. (2006). Global prevalence of dementia: a Delphi consensus study. Tijdschr. Verpleeghuisgeneeskd. 31, 46–46. 10.1007/bf03075138 16360788 PMC2850264

[B15] Gillies R. J. Kinahan P. E. Hricak H. (2016). Radiomics: images are more than pictures, they are data. Radiology 278, 563–577. 10.1148/radiol.2015151169 26579733 PMC4734157

[B16] Gonzalez-Escamilla G. Lange C. Teipel S. Buchert R. Grothe M. J. (2017). PETPVE12: an SPM toolbox for partial volume effects correction in brain PET – application to amyloid imaging with AV45-PET. Neuroimage 147, 669–677. 10.1016/j.neuroimage.2016.12.077 28039094

[B17] Han Z. Wei B. Zheng Y. Yin Y. Li K. Li S. (2017). Breast cancer multi-classification from histopathological images with structured deep learning model. Sci. Rep. 7, 1–10. 10.1038/s41598-017-04075-z 28646155 PMC5482871

[B18] He K. Zhang X. Ren S. Sun J. (2016). Deep residual learning for image recognition. Proc. IEEE Comput. Soc. Conf. Comput. Vis. Pattern Recognit. 2016, 770–778. 10.1109/CVPR.2016.9032166560

[B19] Jeyaraj P. R. Samuel Nadar E. R. (2019). Computer-assisted medical image classification for early diagnosis of oral cancer employing deep learning algorithm. J. Cancer Res. Clin. Oncol. 145, 829–837. 10.1007/s00432-018-02834-7 30603908 PMC11810191

[B20] Khosravi P. Kazemi E. Imielinski M. (2018). EBioMedicine deep convolutional neural networks enable discrimination of heterogeneous digital pathology images. EBioMedicine 27, 317–328. 10.1016/j.ebiom.2017.12.026 29292031 PMC5828543

[B21] Krizhevsky A. Sutskever I. Hinton G. E. (2017). ImageNet classification with deep convolutional neural networks. Commun. ACM 60, 84–90. 10.1145/3065386

[B22] Lange C. Suppa P. Frings L. Brenner W. Spies L. Buchert R. (2015). Optimization of statistical single subject analysis of brain FDG PET for the prognosis of mild cognitive impairment-to-Alzheimer's disease conversion. J. Alzheimers Dis. 49, 945–959. 10.3233/JAD-150814 26577523

[B23] Liu K. Chen K. Yao L. Guo X. (2017). Prediction of mild cognitive impairment conversion using a combination of independent component analysis and the cox model. Front. Hum. Neurosci. 11, 33. 10.3389/fnhum.2017.00033 28220065 PMC5292818

[B24] Lu D. Popuri K. Ding G. W. Balachandar R. Beg M. F. (2018a). Multiscale deep neural network based analysis of FDG-PET images for the early diagnosis of Alzheimer's disease. Med. Image Anal. 46, 26–34. 10.1016/j.media.2018.02.002 29502031

[B25] Lu D. Popuri K. Ding G. W. Balachandar R. Beg M. F. Weiner M.. (2018b). Multimodal and multiscale deep neural networks for the early diagnosis of Alzheimer's disease using structural MR and FDG-PET images. Sci. Rep. 8, 1–13. 10.1038/s41598-018-22871-z 29632364 PMC5890270

[B26] McKhann G. M. Knopman D. S. Chertkow H. Hyman B. T. Jack C. R. Kawas C. H.. (2011). The diagnosis of dementia due to Alzheimer's disease: recommendations from the National Institute on Aging-Alzheimer's Association workgroups on diagnostic guidelines for Alzheimer's disease. Alzheimer's Dement. 7, 263–269. 10.1016/j.jalz.2011.03.005 21514250 PMC3312024

[B27] Moradi E. Pepe A. Gaser C. Huttunen H. Tohka J. (2015). Machine learning framework for early MRI-based Alzheimer's conversion prediction in MCI subjects. Neuroimage 104, 398–412. 10.1016/j.neuroimage.2014.10.002 25312773 PMC5957071

[B28] Pagani M. Nobili F. Morbelli S. Arnaldi D. Giuliani A. Öberg J.. (2017). Early identification of MCI converting to AD: a FDG PET study. Eur. J. Nucl. Med. Mol. Imaging 44, 2042–2052. 10.1007/s00259-017-3761-x 28664464

[B29] Pan X. Phan T-L. Adel M. Fossati C. Gaidon T. Wojak J.. (2021). Multi-view separable pyramid network for AD prediction at MCI stage by 18F-FDG brain PET imaging. IEEE Trans. Med. Imaging 40, 81–92. 10.1109/TMI.2020.3022591 32894711

[B30] Rehman A. Naz S. Razzak M. I. Akram F. Imran M. (2020). A deep learning-based framework for automatic brain tumors classification using transfer learning. Circuits Syst. Signal Process. 39, 757–775. 10.1007/s00034-019-01246-3

[B31] Richard E. Schmand B. A. Eikelenboom P. Van Gool W. A. (2013). MRI and cerebrospinal fluid biomarkers for predicting progression to Alzheimer's disease in patients with mild cognitive impairment: a diagnostic accuracy study. BMJ Open 3, e002541. 10.1136/bmjopen-2012-002541 23794572 PMC3686215

[B32] Roy S. S. Sikaria R. Susan A. (2020). A deep learning based CNN approach on MRI for Alzheimer's disease detection. Intell. Decis. Technol. 13, 495–505. 10.3233/idt-19000533937437

[B33] Sanford A. M. (2017). Mild cognitive impairment. Clin. Geriatr. Med. 33, 325–337. 10.1016/j.cger.2017.02.005 28689566

[B34] Schneider J. A. Arvanitakis Z. Leurgans S. E. Bennett D. A. (2009). The neuropathology of probable Alzheimer disease and mild cognitive impairment. Ann. Neurol. 66, 200–208. 10.1002/ana.21706 19743450 PMC2812870

[B35] Shaffer J. L. Petrella J. R. Sheldon F. C. Choudhury K. R. Calhoun V. D. Edward Coleman R.. (2013). Predicting cognitive decline in subjects at risk for Alzheimer disease by using combined cerebrospinal fluid, MR imaging, and PET biomarkers. Radiology 266, 583–591. 10.1148/radiol.12120010 23232293 PMC3558874

[B36] Spasov S. Passamonti L. Duggento A. Liò P. Toschi N. (2019a). A parameter-efficient deep learning approach to predict conversion from mild cognitive impairment to Alzheimer's disease. Neuroimage 189, 276–287. 10.1016/j.neuroimage.2019.01.031 30654174

[B37] Spasov S. Passamonti L. Duggento A. Liò P. Toschi N. (2019b). A parameter-efficient deep learning approach to predict conversion from mild cognitive impairment to Alzheimer's disease. Neuroimage 189, 276–287. 10.1016/j.neuroimage.2019.1.031 30654174

[B38] Szegedy C. Liu W. Jia Y. Sermanet P. Reed S. Anguelov D.. (2015). Going deeper with convolutions, in 2015 IEEE Conference on Computer Vision and Pattern Recognition (CVPR) (Boston, MA), 1–9. 10.1109/CVPR.2015.7298594

[B39] Vos S. van Rossum I. Burns L. Knol D. Scheltens P. Soininen H.. (2012). Test sequence of CSF and MRI biomarkers for prediction of AD in subjects with MCI. Neurobiol. Aging 33, 2272–2281. 10.1016/j.neurobiolaging.2011.12.017 22264648

[B40] Wang K. Lu X. Zhou H. Gao Y. Zheng J. Tong M.. (2019a). Deep learning radiomics of shear wave elastography significantly improved diagnostic performance for assessing liver fibrosis in chronic hepatitis B: a prospective multicentre study. Gut 68, 729–741. 10.1136/gutjnl-2018-316204 29730602 PMC6580779

[B41] Wang M. Jiang J. H. Yan Z. Z. Alberts I. Ge J. J. Zhang H. W.. (2020). Individual brain metabolic connectome indicator based on Kullback-Leibler Divergence Similarity Estimation predicts progression from mild cognitive impairment to Alzheimer's dementia. Eur. J. Nucl. Med. Mol. Imaging 47, 2753–2764. 10.1007/s00259-020-04814-x 32318784 PMC7567735

[B42] Wang S. H. Xie S. Chen X. Guttery D. S. Tang C. Sun J.. (2019b). Alcoholism identification based on an Alexnet transfer learning model. Front. Psychiatry 10, 205. 10.3389/fpsyt.2019.00205 31031657 PMC6470295

[B43] Yee E. Popuri K. Beg M. F. (2020). Quantifying brain metabolism from FDG-PET images into a probability of Alzheimer's dementia score. Hum. Brain Mapp. 41, 5–16. 10.1002/hbm.24783 31507022 PMC7268066

[B44] Young J. Modat M. Cardoso M. J. Mendelson A. Cash D. Ourselin S. (2013). Accurate multimodal probabilistic prediction of conversion to Alzheimer's disease in patients with mild cognitive impairment. NeuroImage Clin. 2, 735–745. 10.1016/j.nicl.2013.05.004 24179825 PMC3777690

[B45] Zheng X. Yao Z. Huang Y. Yu Y. Wang Y. Liu Y.. (2020). Deep learning radiomics can predict axillary lymph node status in early-stage breast cancer. Nat. Commun. 11, 1–9. 10.1038/s41467-020-15027-z 32144248 PMC7060275

[B46] Zhou H. Jiang J. Lu J. Wang M. Zhang H. Zuo C. (2019). Dual-model radiomic biomarkers predict development of mild cognitive impairment progression to Alzheimer's disease. Front. Neurosci. 13, 1045. 10.3389/fnins.2018.01045 30686995 PMC6338093

