# Supplementary material for: Deep-Learning Radiomics for Discrimination Conversion of Alzheimer's Disease in Patients With Mild Cognitive Impairment: A Study Based on 18F-FDG PET Imaging
Source: Front Aging Neurosci. 2021 Oct 26;13:764872. doi: 10.3389/fnagi.2021.764872 (PMC8576572; doi:10.3389/fnagi.2021.764872)
Supplement: Supplementary file 1 [file Data_Sheet_1.docx]

Supplementary Material

# Supplementary Data

**S1. Radiomic features**

Radiomics analysis was based on a published radiomics tool developed by Vallières et al(Vallières et al., 2015) (https://github.com/mvallieres/radiomics). A large number of texture features extraction and wavelet band-pass filtering algorithms were implemented in this tool. Wavelet band-pass filtering could decompose the ROIs of each image into different wavelet domains. We applied different weights to bandpass sub-bands (LHL, LHH, LLH, HLL, HHL, and HLH) of the ROIs, compared to low- and high-frequency sub-bands (LLL and HHH) in the wavelet domain. The ratio of the weight was defined by R and the values of R were 1/2, 2/3, 1(no wavelet filtering), and 3/2. Subsequently, in each wavelet domain (R = 1/2, 2/3, 1, and 3/2), a total of 43 texture features were extracted using 3D analysis for each individual: 3 histogram-based textures, 9 texture features from the Gray-Level Co-occurrence Matrix (GLCM), 13 texture features from the Gray-Level Run-Length Matrix (GLRLM), 13 texture features from the Gray-Level Size Zone Matrix (GLSZM), and 5 texture features from the Neighborhood Gray-Tone Difference Matrix (NGTDM) (Supplementary Table S1 provides a list of radiomic texture features). The details of these procedures were previously described in Vallières et al. Finally, a total of 172 radiomic features were extracted for each individual.

We extracted 172 high-order quantitative features by radiomics analysis from the corresponding MCI conversion related ROIs for each individual.We constructed the Cox model based on the LASSO-penalized algorithm. And C-index was used to evaluate the prediction accuracy of corresponding Cox model.

**S2. SUVR method**

In the clinical diagnosis and scientific research on PET and MRI, the voxels based method is very common and it plays a specific role in disease assessment. In this study, we introduced mean voxels of 90 regions of interest, which are defined initial brain regions using the Automatic Anatomical Labelling (AAL) template in the Montreal Neurological Institute (MNI) standard space. Image Data from all 355 subjects were utilized to calculate SUVR mean generated the brain regions, by using the whole brain as the reference regions. Te formula to form SUVR mean is

$$SUVR mean= \frac{I_{avg-ROIC}}{I_{avg-ref}}$$

where $I_{avg-ROIC}$ is the average intensity of the brain regions and $I_{avg-ref}$is the average intensity of the reference region.
